# Beta power encodes contextual estimates of temporal event probability in the human brain

**DOI:** 10.1371/journal.pone.0222420

**Published:** 2019-09-26

**Authors:** Alessandro Tavano, Erich Schröger, Sonja A. Kotz

**Affiliations:** 1 BioCog, Cognitive Incl. Biological Psychology, Institute of Psychology, University of Leipzig, Leipzig, Germany; 2 Department of Neuroscience, Max Planck Institute for Empirical Aesthetics, Frankfurt am Main, Germany; 3 Department of Neuropsychology, Max-Planck-Institute for Human Cognitive and Brain Sciences, Leipzig, Germany; 4 Faculty of Psychology and Neuroscience, Department of Neuropsychology and Psychopharmacology, Maastricht University, Maastricht, The Netherlands; Universidad de Salamanca, SPAIN

## Abstract

To prepare for an impending event of unknown temporal distribution, humans internally increase the perceived probability of event onset as time elapses. This effect is termed the hazard rate of events. We tested how the neural encoding of hazard rate changes by providing human participants with prior information on temporal event probability. We recorded behavioral and electroencephalographic (EEG) data while participants listened to continuously repeating five-tone sequences, composed of four standard tones followed by a non-target deviant tone, delivered at slow (1.6 Hz) or fast (4 Hz) rates. The task was to detect a rare target tone, which equiprobably appeared at either position two, three or four of the repeating sequence. In this design, potential target position acts as a proxy for elapsed time. For participants uninformed about the target’s distribution, elapsed time to uncertain target onset increased response speed, displaying a significant hazard rate effect at both slow and fast stimulus rates. However, only in fast sequences did prior information about the target’s temporal distribution interact with elapsed time, suppressing the hazard rate. Importantly, in the fast, uninformed condition pre-stimulus power synchronization in the beta band (Beta 1, 15–19 Hz) predicted the hazard rate of response times. Prior information suppressed pre-stimulus power synchronization in the same band, while still significantly predicting response times. We conclude that Beta 1 power does not simply encode the hazard rate, but—more generally—internal estimates of temporal event probability based upon contextual information.

## Introduction

How do humans successfully prepare for future event onset? When uninformed about the event’s temporal distribution, attention mounts with elapsed time, concurrently increasing response speed and accuracy at event onset [1–17]. This time-dependent performance gain seems to depend on a time-dependent accrual in perceived event probability, termed the *hazard rate of events*. The hazard rate is thought to be endogenously generated by normalizing implicit estimates of target probability (*probability density*), by the ever-diminishing, time-dependent probability that the event has not yet occurred (*survival probability)* [12, 18].

Neural circuits are sensitive to the hazard rate. Shadlen and colleagues [10, 19] showed that neurons in the macaque Lateral Intraparietal Area (LIP) maintain a neocortical representation of the hazard rate. Firing rates in LIP neurons increase with elapsed time irrespective of whether a motor response is given ([19–20]; for a similar result in monkey prefrontal cortex, [21]). In humans, hazard rate effects on cortical activity have been found in regions homologous to the LIP area (Intraparietal Sulcus, IPS: [22–24]; Inferior Parietal Cortex, IPC, [3]), but also in motor, sensory, and prefrontal regions (Supplementary Motor Area, SMA, and the right Superior Temporal Gyrus, STG, for auditory stimuli, 8; premotor cortex, [25]), suggesting the existence of a distributed brain network encoding the hazard rate [5].

**Fig 1:**
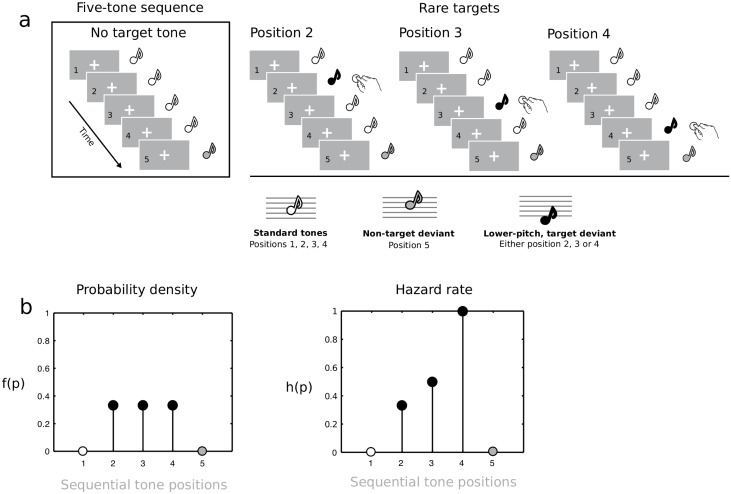
Experimental design. A: Continuously repeating five-tone sequences (left panel, four A_4_ tones followed by a B_4_ tone) were rarely (20% of the times) interspersed with sequences containing a target tone (F_4_) at either of three, equally probable, positions (position 2, 3, and 4, right panel). Elapsed time refers to within-sequence effects of target onset at positions 2, 3 or 4. B: Left, the ensuing discrete probability density function: all potential target onset positions have the same probability. Right, the resulting hazard rate model. The first standard tone and the last non-target tone never hosted a target.

We asked how the hazard rate and its neural underpinnings change when participants are informed about the temporal distribution of an event. To this end, we collected human behavioral and electroencephalographic (EEG) data while participants detected an auditory target events which rarely occurred at different positions within a continuous repeating, five-tone sound sequence. Optimal performance required participants to cycle through potential target onset positions in preparing to respond, in case a target did eventually onset. Humans typically build temporal expectations for uncertain future events. For example, at any given time a soccer player may or may not receive the ball, but she still needs to update her probability estimates about when the ball will be kicked in order to be ready to catch it. The repeating sequence was composed of four standard tones (440 Hz) and a final higher-pitch one (494 Hz; for an overview of the experimental paradigm, see [26–27]). A rare low-pitch target tone (349 Hz) appeared only in 20% of the sequences, substituting a standard tone at either position two, three, or four, with equal probability (Fig 1A and Experimental design). In this design, elapsed time is represented by the within-sequence isochronous unfolding of potential event onset positions within each repeating tone sequence. We expected participants to update target probability estimates within each sequential cycle and response times to reflect a hazard rate distribution (Fig 1B). An increase in perceived temporal probability estimates should also boost early neural sensory processes for events that eventually occur [28–33] as well as late attention-capturing responses [34–39].

However, if prior information is provided to participants about target probability across positions within a sequence, the hazard rate should not be generated as participants would not need to build the survival probability to respond, as simply relying on prior information (equiprobable distribution) would suffice. Our experiment was organized into two sessions. In a first session, participants were uninformed about the target’s distribution. In a second session, participants were informed about the equiprobable target distribution within each repeating sequence. Importantly, in both sessions stimulus sequences were delivered using two different rhythms: first slow (1.66 Hz, resulting from a constant 750 ms Stimulus-Onset-Asynchrony, SOA), and then fast (4 Hz, 250 ms SOA). We hypothesized that fast sequences should favor the perception of five-tone sequences as an auditory object *per se*, making it easier for participants to apply prior information about target position probability.

Recent work suggests that activity in the beta band (13–30 Hz) is a likely candidate for the neural encoding of event probability estimates. Beta-band activity tracks the temporal regularity of isochronous tone sequences [40–42] and correlates with behavioral accuracy [43–45]. In purely perceptual tasks, beta-band power synchronizes [46] with the immediate onset a stimulus [40–41]. Engel and Fries [47] first framed a role for beta-band oscillations in endogenously registering the maintenance or change of a current internal state via cortical feedback projections [48–52]. While previous work analyzed power across the whole beta band, three independent lines of evidence point to a specific role of the low beta-band range, also termed Beta 1 rhythm (< 20 Hz) in perception: 1) Simulation work on neural activity across cortical layers suggests a role for Beta 1 oscillations in distinguishing between novel and standard events [51]; 2) Motor output inhibition has been more frequently linked to high beta-band or Beta 2 activity (> 20 Hz) [52]; 3) Low beta-band (~15 Hz) activity of basal ganglia origin appears to encode an event’s task relevance (target vs. non-target) regardless of motor output [53].

Results show that in fast sequences lower beta band power (Beta 1 rhythm, 15–19 Hz) encodes the hazard rate of events. Providing prior information on target probability suppressed Beta 1 activity as well as the hazard rate of response time, hence reflecting the discrete uniform distribution of targets. Crucially, suppressed Beta 1 power still predicted behavior, suggesting that it does not simply encode the hazard rate, but more generally context-specific temporal probability estimates.

## Results

Twenty-six young adults participated in the experiment, which was organized according to a 2 x 2 design with factors *prior information* and *Stimulus Onset Asynchrony* (SOA). Prior information tested the effect of providing participants with explicit information about the distribution of target tones, while SOA tested whether the effects of presentation rate on extracting stimulus statistics [32–33]. In a first session (*uninformed*), participants were asked to detect the low pitch tone (target) with tones delivered first at a *slow* rate (constant SOA = 750 ms) and then at a *fast* rate (constant SOA = 250 ms). In a second session (*informed*), participants were explicitly informed about the repeating sequence structure and the target’s equal probability distribution across standard positions two, three and four. Each participant received four conditions in the following order: 1) *slow uninformed*; 2) *fast uninformed*; 3) *slow informed*; 4) *fast informed*.

### The hazard rate of response times

**Fig 2:**
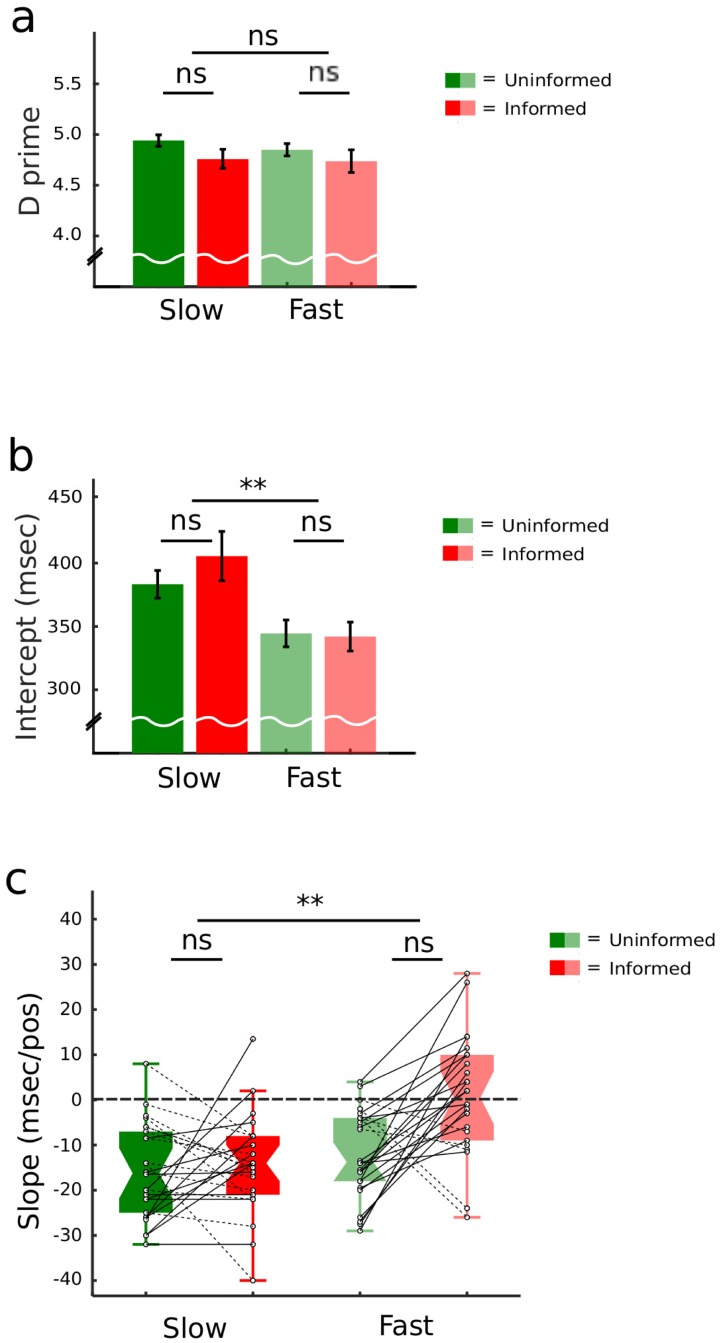
Behavioral results. A: Accuracy as reflected by target detection sensitivity measures (collapsed across potential target position). Error bars represent the Standard Error of the Mean (SEM). B: Means ± SEM of estimated response time intercepts across potential target positions, showing no statistically significant difference among conditions. C: Boxplot with median (middle of each box), interquartile range and whiskers (1.5 time the interquartile range) of estimated response time slopes suggesting the presence of the hazard rate driving response speed in slow stimulus trains, regardless of prior information (participants being uninformed or informed about actual target probabilities), and in fast stimulus trains with uninformed participants. Providing prior information in fast stimulus trains effectively cancelled time-dependent (position-wise) changes in response time, that is, the hazard rate.

Accuracy in target detection, as reflected by task sensitivity (d’) measures, was very high and did not differ among conditions (all *Fs*_(1,25)_ ≤ 2.82, all *ps* ≥ 0.12; Fig 2A). To analyze response times, we fitted nonparametric Theil-Sen estimators [54–55] to single-trial response times across successive potential target positions, our predictor variable. The Theil-Sen estimator is the median of all the lines connecting two data points, and is more robust to outliers as compared to least-squares fit. There resulted one intercept and one regression coefficient (or slope) per participant/condition. Intercept estimates reflect changes in response speed magnitude unrelated to elapsed time, while regression coefficients specifically partial out the effects of elapsed time. The analysis of intercepts showed that participants were overall quicker at responding to targets appearing within faster rather than slower sequences (*F*_(1,25)_ = 40.25, *p* < 0.001; Fig 2B), suggesting a generic facilitation effect for target processing in faster sequences, but we found no effect of prior information and no interaction (all *Fs*_(1,25)_ ≤ 1.12, all *ps* ≥ 0.30). The average response speed for fast sequences was 348 ms (Standard Error of the Mean, SEM = 22), and 411 ms for slow sequences (SEM = 36).

Slopes convey time-dependent changes in response speed using a signed value: a negative value indexes the presence of the hazard rate, as response latencies *decrease* with elapsed time. Slopes distribution was normal in all conditions (all Shapiro-Wilks Statistics ≥ 0.84, all *ps* > 0.21). We found a significant *prior information* × *SOA* interaction (*F*_(1,25)_ = 4.73, *p* < 0.05) driven by a significant effect of prior information in fast sequences (*t*_(1,25)_ = -3.33, *p* < 0.01, all pairwise t-tests fdr-corrected, here and in the following): the hazard rate was present in the fast uninformed condition (*t*_(1,25)_ = 0.05, *p* = 0.95), but not in the fast informed condition (*t*_(1,25)_ = 0.05, *p* = 0.95). The hazard rate was present for both slow conditions regardless of information status (all *ts*_(1,25)_ ≤ -6.06, all *ps* < 0.001). Overall, when significant, the hazard rate effect resulted in a speed gain between 10 and 15 ms per potential target position (Fig 2C).

The suppression of the hazard rate effect by prior information in fast sequences might also have been caused to the cancelling out of random behavioral patterns at the group level, rather than to individually consistent cognitive changes. To verify whether that was the case, we subtracted slope estimates obtained in the uninformed condition from those obtained in the informed condition at either SOA level, and tested the observed data against the null hypothesis that prior information was equally likely to suppress or enhance the hazard rate. A binomial test indicated that in fast sequences prior information suppressed the hazard rate in 20 out 26 participants, that is, in 77% of our sample (*p* < 0.01, Fig 2C), suggesting a non-random effect.

### The hazard rate of sensory processing

First, we verified whether attention had been effectively captured by the five-tone sequence cycle. We hypothesized that if participants perceived non-target tones as the last of each sequence, their onset should have elicited a Mismatch Negativity response signaling pitch-change detection: this hypothesis was verified in all conditions (all *ps* < 0.05, see S1A Fig), suggesting that the non-target deviant effectively reset attention at the end of each five-tone sequence.

We then analyzed the components of the event-related potentials reflecting the elapsed-time component of target onset. A single-trial Theil-Sen regression was run for each time sample at electrode level (epoch duration: 750 ms, including 250-ms pre-stimulus time) and repeated for each participant and condition. There resulted event-related regressions coefficients (ERRCs), which encode the effect of elapsed time on event-related potentials as a difference wave across potential target position, and event-related intercepts (ERIs), which instead encode brain activity unrelated to the passing of time, on a pair with the behavioral analysis [56].

We ran a hypothesis-free, cluster permutation analysis [57] condition-wise to determine the presence of significant activation relative to a surrogate distribution obtained by randomizing data points along the time axis, and then analyzed the effect of prior information by contrasting informed and uninformed conditions within each SOA level. ERIs showed significant characteristic peaks of activity labelled as CNV (Contingent Negative Variation; [25]), N1, N2b, and P3 in all conditions (all *ps* < 0.001). Stimulus rate and prior information resulted in larger N2b/P3 deflections for slower vs. faster sequences, and uninformed vs. informed conditions, respectively (all *ps* < 0.01, S1B Fig; [35]). This confirms previous findings that increasing perceptual expectations reduces surprise-related attentive sound processing.

**Fig 3:**
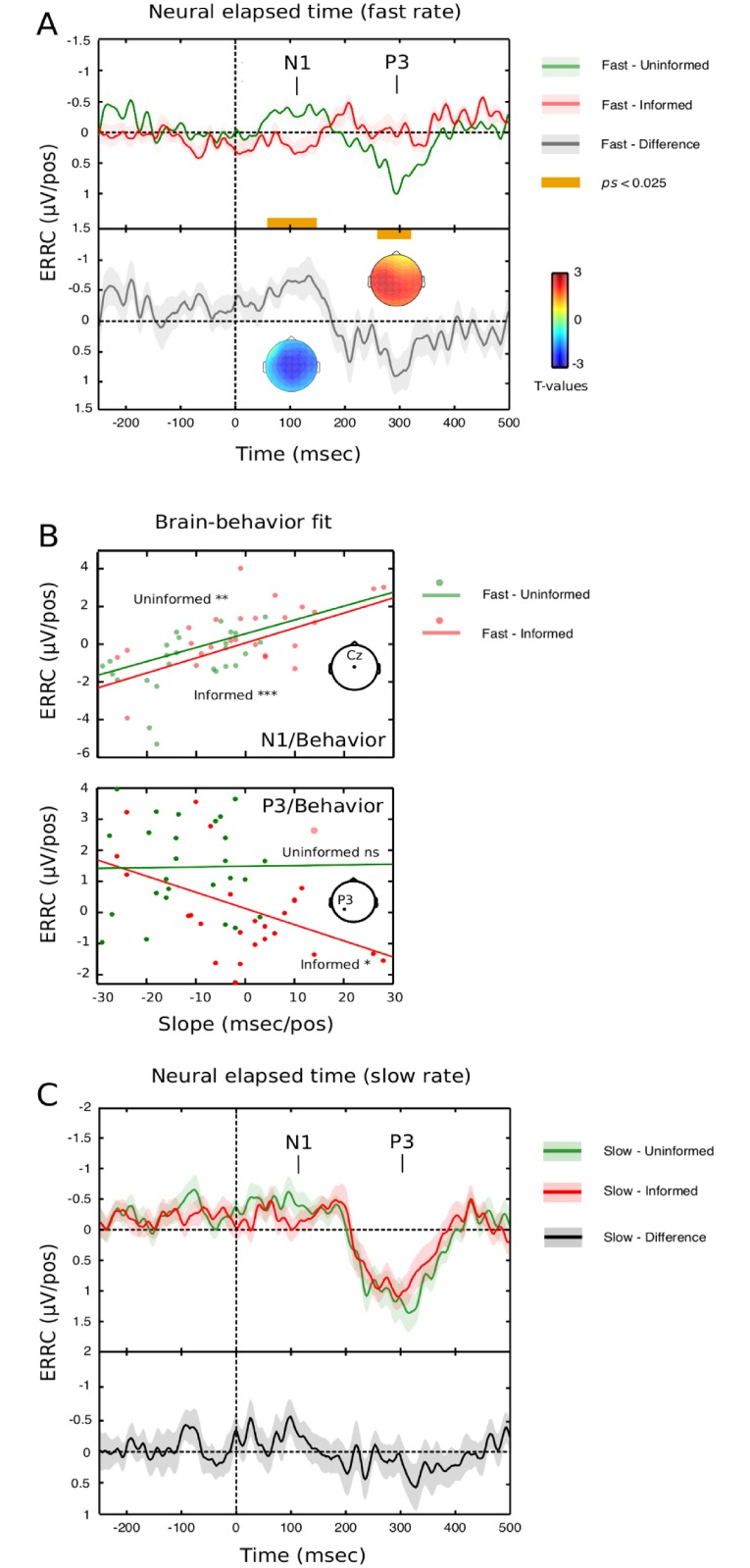
The hazard rate of sensory processing. In the uninformed, fast condition, significant deflections in ERRC activity were found at N1 and P3 latencies. There was no significant activity in the informed condition for the first ~400 ms after target onset. ERRC cluster statistic values averaged across the electrode space show a significant effect of prior information at N1 and P3 latencies, with a central distribution for the negative cluster and a left-sided centroparietal distribution for the positive cluster, consistent with the N1 and P3 interpretations. B. ERRCs at N1 latency in fast sequences significantly predict behavior regardless of prior information; this link is preserved for later, attentive processing (P3) in the informed condition. C. ERRCs in slow sequences show significant ERRC activity at N1 in the uninformed condition, and at P3 latencies in either information condition, highlighting a hazard rate effect of late attentive processing. No statistically significant effect of prior information was found.

As for ERRCs, we first looked at the fast, uninformed condition. We found significant N1 and P3 deflections, with a characteristic topography [58] (*p* < 0.001; Fig 3A, upper panel), and a significant late negativity cluster in the informed condition (> 410 ms post onset, p = 0.05, not shown). When testing the effects of prior information, two significant clusters emerged again at N1 and P3 latencies (Fig 3A, lower panel).

We found a larger N1 deflection (cluster latency 58–156 ms, *p* = 0.018), and a larger P3 deflection in the uninformed condition (cluster latency 257–322 ms, *p* = 0.016). Notably, N1 amplitude significantly predicted response time slopes regardless of information status (uninformed, *rho* = 0.51, *p* < 0.01; informed, *rho* = 0.61, *p* = 0.001, Steiger’s *Z* = -0.52, *p* > 0.60, Fig 3B, upper panel), while P3 amplitudes predicted behavior only in the informed condition (uninformed, *rho* = 0.01, *p* = 0.99; informed, *rho* = 0.43, *p* < 0.05, Fig 3B, lower panel). A topographical analysis (TANOVA, [59]) showed that prior information attenuated P3 activity without changing the underlying generator configuration, while N1 suppression likely depended on changing neural generator between 55 and 100 ms post target onset (S1C Fig). We conclude that for fast sequences, elapsed time enhanced neural afferent activity (N1 wave, [60]) for uninformed participants, thereby increasing response speed. Factoring out elapsed time via prior information correlatively reduced sensory processing and response speed.

ERRCs in slow sequences showed significant brain activity in the P3 range regardless of information status (all *ps* < 0.001), while the N1 deflection was significant only in the uninformed condition (*p* < 0.05, informed: p = 0.28; see Fig 3C). However, we found no significant prior information effect (p = 0.23). P3 ERRCs significantly predicted response time slopes regardless of information status (all *rhos* > 0.48, *ps* < 0.01). Thus, EEG and behavioral data concur to suggest that the down effects of prior information are more likely to be detected using faster stimulus rates [61].

### The hazard rate of neural power

We then focused the analysis of EEG power in sequences. The Theil-Sen estimator was applied to each time-frequency bin, obtaining a distribution of signed time-frequency regression coefficients (TFRCs), measured as μV^2^ unit change per potential target position with negative/positive sign equaling time-dependent decrease/increase. A rank correlation analysis was run between each TFRC bin and response time slopes for the entire epoch (from 250 ms pre-stimulus to 500 ms post-stimulus), in order to isolate the task-relevant components of spectral power.

**Fig 4:**
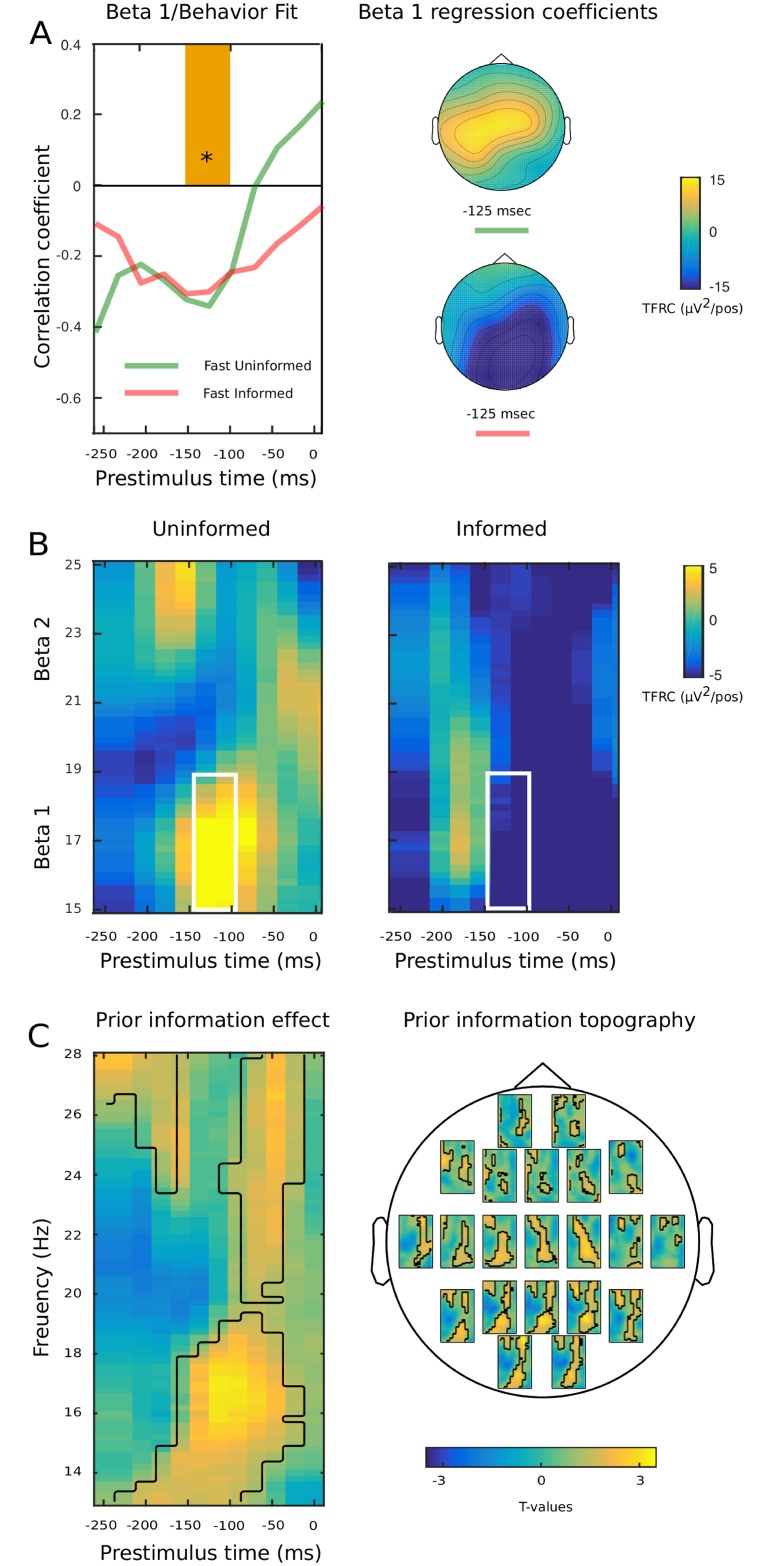
Oscillatory hazard rate in fast sequences. A. Left panel, pre-stimulus Beta 1 (14–19 Hz) oscillatory power (median across all scalp electrodes) significantly predicts response speed to target onset, for both uninformed and informed conditions. Right panel, topography of Time-Frequency Regression Coefficients (TFRCs, measured in μV^2^ per potential target position, median across Beta 1 frequencies) at the behaviorally significant pre-stimulus interval. In the uninformed condition, the hazard rate is reflected at central electrodes; in the informed condition, prior information about actual target onset cancels the hazard rate at central electrodes and leads to a significant desynchronization at parietal electrodes. B. Grand median time-frequency representation of oscillatory power regression coefficient distribution at both Beta 1 and Beta 2 in uninformed (right panel) and informed (left panel) fast sequences. White squares indicate the time-frequency bins averaged to obtain the B1/behavior fit in 4A. C. Left panel, cluster-based significance distribution of prior information across beta-band frequencies at Ps electrode. The center of gravity lies in the Beta 1 rhythm range, between 150 and 50 ms before the eventual onset of the uncertain target: These bins are fully included in the independently identified behaviorally significant pre-stimulus interval. Right panel, topography of the effect of prior information.

In the following, we focus on fast sequences in order to highlight the effect prior information. For participants who were uninformed about the target’s temporal distribution, we expected single-trial power in the Beta 1 range (< 20 Hz) to increase with elapsed time, encoding the task relevance of a potential target onset. We measured the power spectrum of each trial epoch, from 5 to 28 Hz. A concentration in Beta 1 power (15–19 Hz, averaged across all scalp electrodes) at a latency of about -150 ms (pre-stimulus baseline), inversely correlated with response time slopes (*p* < 0.05, Fig 4A, left panel). Notice that, by design, participants could not foresee whether a target would appear or not at any potential position within the current sequence. This suggests that synchrony in the beta band encodes the update of time-dependent probability estimates for potential future events, driving an increase in response speed for targets which eventually did appear. Beta 1 power synchrony was significant at left centroparietal electrodes (*p* < 0.05, Fig 4A, right panel). Importantly, Beta 1 power also predicted post-stimulus ERRC N1 amplitudes (*p* = 0.01), establishing for the first time a direct connection between pre-stimulus probability update and post-stimulus sensory processing.

With prior information, probability update processes in the Beta 1 band were suppressed at central electrodes, and a significant decrease in spectral power was found at parietal electrodes (*p* < 0.05, Fig 4A, right panel). This may reflect how the brain silences the hazard rate at sensory-specific central electrodes. Notably, the correlation of Beta 1 power with response time slopes remained significant (*p* < 0.05, average across all scalp electrodes, Fig 4A, left panel). Similarly, Beta 1 power maintained a significant correlation with post-stimulus N1 amplitudes (*p* < 0.05), confirming the inference of a causal link between pre-stimulus Beta 1 power and sensory processing. We found no significant correlation with response times for either Alpha (8–12 Hz, all *ps* > 0.2) or Beta 2 (21–25 Hz, all *ps* > 0.08) bands, suggesting the encoding of perceived event probability was specific to Beta 1 rhythm.

## Discussion

To prepare for future events, humans could simply estimate the probability density function of target onset over trials and use that information to respond [62, 63]. However, “that is not the natural way one thinks about it as the [waiting] process unfolds in time. Rather, if the event has not yet occurred, one senses there is some tendency for it to occur the next instant in time” [10,12,19]. We found that such a “feeling” is present also for target events that may or may not onset within a given interval. Participants naïve to target distribution probabilities used elapsed time to progressively increase their temporal expectations about potential target onset, regardless of whether stimulus rate was slow or fast. However, when informed about the equiprobable potential target onset within each five-tone sequence, a suppression effect of prior information on the hazard rate was detected in fast sequences only. We ascribe the absence of a prior information effect in slow sequences to a difficulty for participants to represent five-tone sequences as an integrated percept within sensory memory [26–27, 64–66]. Notice that the attenuation of the hazard rate in fast sequences was not due to increased variance at group level but rather to suppressive processes acting at individual level (in ~ 77% of our sample). We suggest that prior information factored out elapsed-time dependent processes, such as the calculation of survival probability, from the computation of position-wise target probability. However, it is important to underlie that the discreet uniform probability density, which is assumed to be endogenously computed by participants, coincides with the distribution highlighted by prior information, so at present their contribution to the effect prior information on response times in fast sequences indistinguishable.

We found an increase in early post-stimulus sensory activity in the N1 range in the uninformed condition in fast sequences. This increase in brain activity explained a significant proportion of individual level variance in response speed, suggesting that modulations of N1 coefficients encode time-dependent information. However, prior information significantly suppressed the N1 deflection, while remarkably maintaining a consistent predictive relationship to response behavior, suggesting that encoding temporal probability changes the weights of sensory processing.

Low beta-band power (Beta 1 = 15–19 Hz), ~150–125 ms before the potential onset of the target event, consistently predicted the effect of elapsed time on response speed and sensory processing in both uninformed and informed fast sequence conditions. For uninformed participants, with elapsed time factored in, Beta 1 increased at central electrodes. When elapsed time was factored out by prior information, Beta 1 did not change at central electrodes and was significant suppressed at parietal electrodes. The latter result could reflect how a distributed neural system silences the sensory effects of elapsed time.

The finding of a Beta 1 power modulation as a neural correlate of the hazard rate expands on the original proposal of Engel and Fries [50], as well as the findings of Arnal et al. [43] and Fujioka et al. [40–41], by demonstrating that beta power does not simply reflect temporal tracking of event onset, but rather subjective estimates of temporal probability. Todorovic and colleagues [67] found beta-band desynchronization for expected auditory events, but only in a passive listening condition, with no difference when attention was deployed on sound sequences. Spitzer and Haegens [50] suggest a primary role of beta band in the endogenous re-activation of neuronal ensembles coding for task-relevant information (see also [48–49, 51]). A potentially fruitful endeavor for future research would be to bridge human and animal research by specifically testing the relationship between cortical Beta 1 power and the encoding of the hazard rate at the neuronal level [10, 19].

The neural mechanisms underlying elapsed time to target under most distribution functions (except exponential ones, also termed memoryless functions, for which no hazard rate is elicited [12]) constitute a particular case of perceptual bias. Participants gain a substantial behavioral advantage by constructing subjective temporal expectations rather than relying on probability density estimates. We showed that providing truthful prior information on the target’s uniform probability density cancels any behavioral advantage in fast sequences. It follows that the hazard rate of events represents a conceptual challenge for active sensing models of perception that construe the fit between brain and behavior as based on precise internal models of actual event statistics, which invariably ascribe a facilitative effect to veridical prior information [28].

Contrary to previous work with deterministic targets within a foreperiod design [68–69], we did not find significant effects of elapsed time in the alpha band. This may be due to the sequential cycling of attention, which may have entrained sub-bands of the alpha rhythm or to the fact that we used an uncertain target. It is also possible that the use of target sounds may have accentuated the relevance of low beta relative to alpha activity [70].

It has been postulated that changes in neural rhythms may underlie different cognitive operations [71]. Notably, simulations studies of cortical rhythm formation postulate that the Beta 1 rhythm reflects memory of stimulation history [51]. The importance of cortical Beta 1 in defining the task-relevance of sensory stimuli could depends on contributions from basal ganglia generator circuits [53]. Interestingly, recent clinical work purports that beta desynchronization in Parkinson’s disease (PD), predominantly of basal ganglia origin, specifically impairs pre-stimulus beta-band activity in rhythmic auditory perception, suggesting a causal relationship with timing deficits that are typically present in PD [72]. This patient group could provide a test case to better analyze the functional specificity of synchronization and desynchronization in the low vs. high beta band, contrasting movement initiation and task relevance [46].

## Conclusions

Modulations of Beta 1 rhythm reflect how participants internally prepare to respond to uncertain events, keeping into account what they know about their temporal distribution.

## Materials and methods

### Participants

The experiment was conducted at the Max Planck Institute for Human and Cognitive Brain Sciences, Leipzig (Germany). Thirty healthy young adult individuals (15 female; age range = 19–31, mean = 25, SD = 3.5) were recruited from the institute’s database of participants. All individuals had university-level education and were paid for their participation. Four participants were excluded from further analysis: two for below-average behavioral performance (less than 50% target detection), one for misinterpreting task instructions, one for excessive number of rejected target epochs (cut-off: 80%, i.e. at least 16 target trials per position and condition). The final pool of 26 participants (13 females) reported no neurological or psychiatric disorders or therapies involving the central nervous system. Individually measured, bilateral audiometric thresholds of at least 30 dB Hearing Level at 0.25–8 KHz octave frequencies [73]. All participants signed a written informed consent complying with the Declaration of Helsinki on human experimentation. This study was approved by the Ethics Committee of the University of Leipzig.

### Stimuli

Stimuli were three 50-ms pure tones (5 ms rise/fall), binaurally presented via loudspeakers at ~80 dB SPL and generated using Matlab (version 7, Mathworks, Natick, MA). A 440 Hz tone (A4 on the equal tempered scale), termed *standard tone*, was presented 900 times per condition (75% global probability). A 494 Hz tone (B4, two semitones higher than the standard), termed *non-target tone*, was presented 240 times per condition (20% global probability). A 349 Hz tone (F4, four semitones lower than the standard), termed *target tone*, was presented 60 times per condition (5% global probability). Fig 1 illustrates stimulus organization. Standard and non-target tones were organized as a continuously repeating sequence of four standard tones followed by a non-target tone always in fifth position. Target tones appeared in 20% of the sequences, randomly at either standard position two, three or four, with a uniform distribution. There was a maximum of one target per sequence, and minimally two successive sequences without targets before a target-containing sequence. Stimulus sequences were delivered using Presentation software (version 12.0, www.neurobs.com) running on a Windows PC.

### Experimental design

Participants sat in an electrically shielded, sound-attenuated chamber, and fixated a white cross on black computer screen at a distance of ~1 meter while listening to the auditory stimuli. They responded to target tone onset by pressing a button on an external response box.

Targets were equiprobably distributed across standard position two, three and four, yielding a discrete uniform distribution function: f(t) = 1/3, for each of standard positions two, three, and four. Denoting the survival probability (“the event has not yet occurred”) as 1 − F(t), where F(t) is the cumulative distribution function, the hazard function is then: h(t) = f(t)/(1-F(t)).

The experiment was organized into two successive sessions, with a fixed order. In the first session, participants were unaware of target distribution and instructed to respond to the onset of target tones as accurately and fast as possible by pressing a button on a response box. They trained in a short block of 60 experimental randomly distributed tone sequences containing three targets. If errors were made (Missing, False Alarm), the training block was repeated until no errors were detected. Experimental tone sequences were first delivered with a constant 750-ms stimulus onset asynchrony (SOA), corresponding to 1.6 Hz stimulus rate (three 5-min blocks; first condition), and then—after a short break—with a constant 250-ms SOA, corresponding to 4 Hz stimulus rate (one 5-min block; second condition). The first two conditions were tagged as *slow uninformed* and *fast uninformed*, respectively. In a second session, after a 15-min break, participants were informed, both verbally and using visual aids, both about the repeating tone sequence and target probability distribution within a sequence. The instruction, therefore, changed: they were asked to respond to target tones whose onset would break the sequence at either standard position two, three, or four. They again trained with a short block of 60 tones and 3 targets. If errors were made (Missing, False Alarm), the training block was repeated until no errors were detected. The experimental tone trains were again first delivered with a 750-ms constant SOA (three 5-min blocks; third condition), and then—after a short break—with a 250-ms constant SOA (one 5-min block; fourth condition). These conditions were tagged as *slow informed* and *fast informed*, respectively. Participants completed the experiment in about one hour.

### Behavioral data analysis

Hits were all button presses whose response time ranged between 150 and 1250 ms from the onset of a target tone. Button presses recorded after 1250 ms were considered as false alarms (FA). Accuracy was measured by z-transforming hit and false alarm counts (5% adjustment for ceiling effects) to obtain a d’ index of task sensitivity (zFA—zHit, [74]). A single-trial linear fit allowed detecting the presence/absence of the hazard rate: slope sign indicates an increase or decrease in response times for long awaited events, while slope magnitude measures the strength of change with elapsed time. The Theil-Sen estimator represents an unbiased, robust, non-parametric simple linear regression method, which extracts the median slope among all possible pairwise combinations of points (54–55). Accuracy and reaction time data entered a two-way, repeated-measures ANOVA, with the factors SOA (slow, fast) and prior information (uninformed, informed). Results with p ≤ 0.05 (fdr-correction was applied to protect against family-wise error rate) were declared significant.

### EEG data acquisition and pre-processing

Electroencephalographic (EEG) data were collected using a 26 scalp Ag/AgCl electrode set (BrainAmp), mounted in an elastic cap according to the 10–20 International system. The electrode space was composed by: Fp1, Fpz, Fp2, F7, F3, Fz, F4, F8, FT7, FC3, FC4, FT8, T7, C3, Cz, C4, T8, CP5, CP6, P7, P3, Pz, P4, P8, O1, O2. Two external electrodes were placed at right and left mastoid sites. For electrooculographic (EOG) data recording, four additional electrodes were placed at both eye canthi, and above and below the right eye. For participants 19 to 26 (21–30 in the original dataset), the cap contained 38 more electrodes (10–10 system), not used in the current analysis for comparability across participants (Fpz was excluded from participants 1–18 as it was conversely not present in participants 19–26). An online reference was placed on the tip of the nose and the sternum served as ground. Electrode impedance was kept below 5 kΩ. EEG/EOG sampling rate was set to 500 Hz, with online high-pass filtering at 0.01 Hz. The resulting continuous recordings were visually inspected and pruned from non-stereotypical artefacts or extreme voltage changes values. An Independent Component Analysis (ICA, extended Infomax, [75]) was performed on the pruned continuous data, offline bandpass filtered 1–100 Hz (Kaiser window, Beta 5.6533, filter order 1812 points, transition bandwidth 1 Hz, see [76]). The maps of exemplar Independent Components (ICs) reflecting blinks or vertical eye movements and horizontal eye movements from one participant were selected as spatial templates in a semi-automatic artefact search across all ICs of the remaining datasets (correlation threshold, *r* = 0.94; [77]). Eye-movement-related ICs, both vertical/blink-related and horizontal, ranged between 1 and 3 per participant. Selected ICs were verified in their spectral power distribution before being subtracted [78]. The resulting continuous datasets were low-pass filtered at 35 Hz (filter order 184, transition bandwidth 10 Hz).

### Event-related potential and coefficient analysis

Epochs were separately extracted for the onset of standard, non-target and target stimuli, beginning 1000 ms before and ending 1000 ms after stimulus onset. Epochs were selected based on their contribution to increasing the signal-to-noise ratio [79]. On average, 12.3% of epochs were rejected. Epochs of interest for regression analysis began 250 ms before the onset of target tone, and ended 500 ms thereafter. Neural responses for non-target tones were calculated in sequences that did not contain a target trial, by subtracting the response of the fourth standard tone. Stimulation was isochronous, so that target position directly reflects elapsed time. For each time point of each Target trial, a Theil-Sen estimate of the linear relationship between target position as predictor and event-related electrical activity was calculated. There resulted event-related regression coefficients (ERRCs, slopes) and event-related intercepts (ERIs). ERRCs encode neural estimates of elapsed time [56, 80]. We took a data-driven approach to analyze the distribution and polarity of neural effects within a post-stimulus window of interest (0–600 ms). The presence of differences in the effects of Target position (elapsed time) as determined by prior information was tested for all channels/time points separately at each SOA level: slow uninformed vs. slow informed; fast uninformed vs. fast informed. ERRCs entered a non-parametric, cluster-based permutation test of significance, testing the effect of prior information at each SOA level [57]. Clusters were minimally composed of two electrodes. Cluster-level statistics was determined by summating significant paired T-test values (cluster alpha = 0.05) across adjacent points within each cluster, and evaluated under the distribution obtained by drawing 1000 within-subject, random permutations of the observed data. Cluster-based tests against zero were run by contrasting each condition with a surrogate distribution obtained by randomizing individual data points along the time axis. Significance was set at p = 0.05. Topographical differences in the distribution of current density were investigated using the Global Dissimilarity Index, which measures the configuration of electric fields (and their linear transformations), normalized by their individual strength [59].

### Time-frequency analysis

To increase signal-to noise ratio, data were subject to a Principal Component Analysis, and the first four components, accounting on average for 93% of variance, were retained for further processing. Zero-padded (5 s), individual target epochs were submitted to a time-frequency analysis at each electrode using a Morlet wavelet (7 cycles, [81]), for frequencies comprised between 5 and 28 Hz, in steps of 0.25 Hz. Event-related power estimates of target position effects at each frequency/time point were extracted from 500 ms pre-stimulus to 500 ms post-stimulus (sliding window = 25 ms). Each time-frequency bin entered a Theil-Sen, non-parametric regression analysis with Target position as predictor, obtaining time-frequency regression coefficients (TFRCs), and time-frequency intercepts (TFIs). We obtained median TFRC power estimates across all electrodes at Alpha (8–12 Hz), Beta 1 (14–19 Hz) and Beta 2 (20–25 Hz) rhythms, and calculated a Kendall-type [82] rank correlation between each TFRC and response time slopes in order to determine which time-frequency band were relevant for behavior. A permutation resampling approach (1000 repetitions) was used to test the significance of rank correlation results.

To analyze the effect of prior information within each SOA level, we again resorted to a cluster-based permutation approach of power estimates between 250 ms pre-stimulus and 500 ms post-stimulus, using paired T-tests (significance set at 0.05, minimal N electrodes per cluster = 1, T-test cluster parameter = maxsize, 1000 permutations, cluster alpha set at 0.1; [57]). All analyses were run using EEGLAB [75], FieldTrip [81], and custom Matlab scripts. The datasets generated during and/or analyzed during the current study are available from the corresponding author on reasonable request for researchers who meet the criteria for access to confidential data of the Max Planck Institute for Human Brain Sciences.

## Supporting information

S1 Fig Attention cycles, event-related intercepts (ERI) and N1-P3 event-related coefficients (ERRCs). We first used brain data to verify whether participants’ attentive searchlight was effectively deployed on individual five-tone sequences during the experiment, as per our assumption. If the fifth, non-target tone reliably indexed the end of each sequence, it would generate a prediction error response relative to the preceding standard, resetting attention to the next incoming sequence. Indeed, we found a significant prediction error response in all conditions (all *ts*_(1,25)_ ≤ -2.69, all *ps* < 0.05), with non-target deviant N1 being more negative than the standard N1 in the fourth position, with no significant difference among conditions (all *Fs*_(1,25)_ ≤ 1.60, all *ps* ≥ 0.21, S1A Fig). Thus, the fifth, non-target tone similarly favored in all condition the extraction of individual five-tone sequences from the attended stream of sounds. Event-related intercepts showed CNV, N1, N2b, and P3 deflections in all conditions (all *ps* < 0.001, S1B Fig). However, prior information suppressed only the N2b component of event-related intercepts in both slow and fast sequences (slow: cluster latency 220–332 ms, *p* = 0.01; fast: cluster latency 172–308 ms, *p* < 0.001; cluster overlap across SOA levels = 78%). Similarly, short SOA suppressed activity for late components in the informed condition (cluster latency > 312 ms, *p* < 0.01). This was further verified by calculating the correlation between N1 and at P3 activity at each scalp electrode for either information condition. A permutation resampling analysis showed that only in the informed condition did deviant N1 activity significantly predict P3 activity (*p* < 0.01; see S1C Fig, upper panel). This led to a significant difference between informed and uninformed conditions (*p* < 0.001). To detect any qualitative difference over and beyond the quantitative changes in the time domain, we measured the degree of dissimilarity between the scalp configurations of the cortical ERRC generators by means of a Topographical Analysis of Variance (TANOVA), which corrects for differences in overall response magnitude at electrode level. We found a significant effect of prior knowledge in two adjacent clusters roughly between 55 and 100 ms post-onset, corresponding to the early deviant N1 deflection (S1C Fig, lower panel).
(EPS)

S1 Text (DOCX)
