# Supplementary material for: Beta power encodes contextual estimates of temporal event probability in the human brain
Source: PLoS One. 2019 Sep 26;14(9):e0222420. doi: 10.1371/journal.pone.0222420 (PMC6762064; doi:10.1371/journal.pone.0222420)
Supplement: S1 Text — (DOCX) [file pone.0222420.s002.docx]

**Supporting information**

**Reaction times**

Mean reaction times were entered in a 3 x 2 x 2 rmANOVA with factors Target Position (Position 2, 3, 4), Prior information (uninformed, informed), Stimulus Onset Asynchrony (slow sequences, fast Sequences).

**Cyclical deployment of attention**

To calculate prediction error brain responses to non-target deviant tones, event-related responses were first re-referenced to the digitally linked mastoids. Then, the response to the standard tone in the fourth position was subtracted from that of non-target deviant tones for sequences that did not contain a target. All significant p-values were fdr-corrected to protect against family-wise error rate.

**Event-Related Intercepts**

Target position (elapsed time) as determined by Prior Information was tested for all channels/time points separately at each SOA level: slow Uninformed vs. slow Informed; fast Uninformed vs. fast Informed. ERRCs entered a non-parametric, cluster-based permutation test of significance, which allows controlling for type I error rate in the presence of multiple comparisons. Clusters were minimally composed of two electrodes. Cluster-level statistics was determined by summating significant paired T-test values (cluster alpha = 0.05) across adjacent points within each cluster, and evaluated under the distribution obtained by drawing 1000 within-subject, random permutations of the observed data. Results show the probability (alpha = 0.05) of obtaining a cluster-level statistic that is larger (positive polarity) or smaller (negative polarity) than the observed one.

**N1-P3 correlation**

We calculated the correlation between N1 and P3 activity for each time-electrode pair, for each information condition in fast sequences. The resulting matrix was then binned and transformed into percent measures to obtain a distribution of correlation coefficients. Then, we took a permutation resampling approach (N = 1000) to estimate both skewness (*p* < 0.01, informed condition), and the difference between informed and uninformed conditions (*p* < 0.001).

**Topographical dissimilarity analysis**

Reference-free, current density maps at electrode level were calculated by computing the second spatial derivative (1-3; maximum degree of Legendre polynomials = 50, order of splines = 4, smoothing = 10e-5). To detect any qualitative difference over and beyond the quantitative changes in brain activity brought about by prior information, we resorted to a Topographical Analysis of Variance (TANOVA), which corrects for differences in overall response magnitude at electrode level. TANOVA is based on the Global Dissimilarity Index, which measures the configuration of electric fields (and their linear transformations, such as the ERRCs), normalized by their individual strength Global Field Power.

**Supporting References**

1. Perrin F, Pernier J, Bertrand O, Echallier JF. Spherical splines for scalp potential and current density mapping. Electroencephalogr Clin Neurophysiol. 1989; 72: 184-187.
2. Perrin F, Bertrand O, Giard MH, Pernier J. Precautions in topographic mapping and in evoked potential map reading. J Clin Neurophysiol. 1990; 7: 498-506.
3. Srinivasan R. High-Resolution EEG: Theory and Practice. In: Handy TC, editor. Event-Related Potentials: A Method Handbook. Cambridge(MA): The MIT Press; 2005. pp. 167-188.
